# Limited opportunity for nursing students to perform practical nursing skills during clinical placements: a Scandinavian cross-sectional study

**DOI:** 10.1016/j.ijnsa.2026.100608

**Published:** 2026-06-24

**Authors:** K Jonsson, K Falk-Brynhildsen, M Ravik, IT Björk, K Blomberg, SE Husebø, IÅ Reierson, I Sommer, K Bölenius

**Affiliations:** a Faculty of Medicine, Department of Nursing, Umeå University, Umeå, Sweden; b Faculty of Medicine and Health, School of Health Sciences, Örebro University, Örebro, Sweden; c Department of Nursing and Health Sciences, University of South-Eastern Norway, Norway; d Department of Public Health Sciences, University of Oslo, Norway; e Faculty of Health Sciences and SHARE, University of Stavanger, Norway; f Department of Cardiology, Aarhus University Hospital, Denmark

**Keywords:** Clinical practice, Clinical placement, Education, Nursing student, Practical nursing skills

## Abstract

**Background:**

Practical nursing skills are complex actions performed daily in nursing practice. Structured learning and repeated practice help students develop clinical competence. Clinical placements are pivotal in learning, with factors such as educational context, preceptor availability, and time constraints shaping students’ learning opportunities.

**Objectives:**

To describe nursing students’ opportunities to perform practical skills during clinical placements.

**Design and Setting:**

A quantitative cross-sectional study conducted in 2023 involved 706 nursing students from Denmark, Norway, and Sweden. Data were collected via a questionnaire and analyzed using descriptive statistics.

**Results:**

Students reported substantial variations in the practical skills performed during clinical placements. Overall, the frequency of skills performance was low across all years and domains, including practical nursing skills concerning respiration, circulation, elimination, nutrition, activity, skin care, and medication. Supervision was generally available, though some skills lacked adequate preceptor involvement.

**Conclusions:**

The study highlights disparities in nursing students’ opportunities to practice essential skills during clinical placements. The lack of practice across a wide range of skills may compromise the quality and thoroughness of nursing education, affecting the students’ professional preparedness.

**What is already known about the topic**

• Practical nursing skills need structured learning and repeated practice.
• Clinical placements are key, but skills practice depends on supervision and context.
• Simulation is common, but curricula vary and transfer to practice is uncertain.

Alt-text: Unlabelled box dummy alt text

**What this paper adds**

• Core nursing skills are underrepresented in clinical practice placements.
• Across Scandinavia, students reported low and uneven clinical skills practice.
• Some students lacked supervised opportunities for specific practical skills.

Alt-text: Unlabelled box dummy alt text

## 1 Introduction

The performance of practical nursing skills is a frequent and fundamental aspect of nursing practice, requiring high accuracy to ensure safe patient care (Ewertsson et al., 2015; Mathisen et al., 2022). To support this, previous research has emphasized the value of hands-on experience and repeated practice in developing nursing students’ clinical competence and practical skills (Song and McCreary, 2020). Consequently, the transmission of practical skills is a cornerstone of nursing education (Loewen et al., 2017), and sharing the responsibility between nursing education programs and healthcare organizations is essential to support this process (Ewertsson et al., 2015). Despite the recognized importance of teaching the accurate performance of practical skills, nursing education programmes lack an agreed-on prioritization of which practical skills should be taught, their appropriate timing across the educational years, and the optimal learning environment for their instruction (clinical or campus-based) (Bölenius et al., 2024).

Simulation centers play a central role in nursing education by providing structured opportunities for students to learn practical skills and build clinical competence ahead of clinical placements (Daneshfar and Moonaghi, 2025; Hilleren et al., 2022; Ravik and Bjørk, 2023; Reierson et al., 2024). Students engage in simulation-based learning experience (SBLE) at various stages of their education, allowing them to gradually build proficiency in a broad set of practical nursing skills, as emphasized in previous studies (Bölenius et al., 2024; Ravik and Bjørk, 2023). However, a Scandinavian study identified wide variation in the types and numbers of practical skills taught across different simulation centers (Bölenius et al., 2024). Supporting this, a narrative review of 121 studies from 26 countries revealed significant variation, with over 50 different practical nursing skills included in curricula and taught in simulation or skills centers (Hilleren et al., 2022). Ensuring that nursing education programs provide sufficient competencies, preparing nursing students to meet the demands of clinical placements, remains a considerable challenge for these programs (Axelsson et al., 2021; Houghton et al., 2013; Ravik et al., 2014; Strobl et al., 2021).

According to EU directives, students are required to complete 90 European Credit Transfer and Accumulation System (ECTS) units of clinical placement. Clinical placement should be supervised by a preceptor, providing students with opportunities to integrate theoretical knowledge with practical skills (European Commission, 2013; Henriksen et al., 2020). Clinical placement is a crucial component of nursing education; however, the effectiveness and quality of practical skills learning can vary significantly depending on numerous factors, including the diversity of educational frameworks, availability of experienced supervisors, and time allocated for the supervisor’s role (Mathisen et al., 2023). Other factors, such as increased workload in healthcare settings and the increased presence in clinical practice of students representing nursing and other healthcare professions, may influence the quality of practical skills practice (Rudland et al., 2025). The growing specialization within healthcare settings often restricts nursing students to practicing only a limited repertoire of practical skills during each clinical placement (Hayden et al., 2014; Södersved Källestedt et al., 2020). Furthermore, the increasingly short duration of patient hospital stays, combined with high levels of patient acuity, may limit nursing students’ opportunities to develop practical nursing skills, as the high-pressure environment reduces time for supervised practice. These conditions often limit the time available for supervised clinical interactions (Hayden et al., 2014). Limited opportunities to learn essential practical nursing skills in clinical placements impede students’ ability to acquire the skills needed for their future roles as registered nurses (Schroers et al., 2023; Waldner and Olson, 2007). Given these many limiting factors, it is crucial to ensure students’ opportunities to practice practical nursing skills during clinical placements, and to ensure a sufficient extent of such training and of the support provided. Understanding these conditions also highlights the pivotal role of the preceptor.

In this paper, we use the term “preceptor” to refer specifically to an experienced registered nurse (RN) who directly supervises and guides nursing students during their clinical placements. This role is distinct from that of nurse educators, who may have broader educational or administrative responsibilities in the academic or clinical setting. The RN preceptors are crucial for practical nursing skill learning, providing supervision and direction during clinical placements (Pramila‐Savukoski et al., 2020; Tuomikoski et al., 2020). Sufficient time for preceptors to be present and create an enabling clinical environment helps students integrate evidence-based knowledge, which is essential for developing effective nursing interventions (Jonsén et al., 2013). Their extensive experience and regular feedback help students refine their skills and build confidence (Ravik et al., 2017). Effective supervision fosters professional identity, confidence, and the development of both technical and non-technical skills necessary for competent practice (Leonardsen et al., 2021). However, a recent study reports that preceptors identified challenges related to student nurses’ practical skill learning during clinical placements, including issues of knowledge, attitudes, engagement, and emotions (Ravik et al., 2025).

The Nordic countries (i.e., Denmark, Finland, Iceland, Norway, and Sweden) maintain close contacts in healthcare and education, and their nursing education courses share similar content and structure. In Norway and Sweden, nursing education requires 180 ECTS and includes a bachelor’s degree in nursing, while in Denmark, nursing education requires 210 ECTS for the same degree (Henriksen et al., 2020).

Although simulation-based learning environments are widely used in nursing education, their effectiveness in preparing students for the practical demands of clinical settings remains uncertain. This is partly due to the lack of consensus regarding which practical skills should be prioritized, when they should be introduced, and in which learning environments they are best taught. Existing research further highlights substantial variability in both the number and types of skills included in curricula and simulation training, with Scandinavian studies demonstrating differences across simulation centers and international evidence identifying a wide range of skills taught across programs. At the same time, ensuring that students achieve sufficient clinical competence remains a key challenge. Structural factors in healthcare settings, such as high workload and increasing numbers of students, may restrict opportunities for supervised practice during clinical placements. Limited access to such opportunities, together with reported challenges from preceptors related to students’ engagement, knowledge, and confidence, suggests a misalignment between educational preparation and clinical expectations. Despite these challenges, there is limited empirical evidence particularly in the Scandinavian context quantifying the extent to which students are offered opportunities to perform practical skills during clinical placements and the degree to which they receive guidance from preceptors.

To address this gap, the present study adopts a quantitative approach to describe nursing students’ opportunities to perform practical nursing skills during clinical placements, specifically examining the extent, frequency, and variation of skill performance, as well as the degree of guidance provided by preceptors. By systematically capturing these dimensions, the study contributes to a more precise understanding of how clinical learning environments support the development of practical competence.

### 1.1 Aim and objectives

The overall aim was to describe nursing students’ opportunities to perform practical nursing skills during clinical placements. The specific research questions were:

− To what extent and how frequently are practical nursing skills practiced during nursing students’ clinical placements?
− To what degree do nursing students receive guidance on practical nursing skill performance from preceptors?

## 2 Method

This study is part of the Skill Practice in Nursing Education (SPiNE) project, which aims to generate new insights into the factors influencing students’ acquisition of practical nursing skills during their bachelor nursing education (Reierson et al., 2024). The SPiNE project is being conducted by the Scandinavian collaborative research group, Research in Nursing Skills (RiNS). This network was established in 2006 and comprises nurses from clinical practice, education, and academia across Denmark, Norway, and Sweden. The group aims to advance knowledge of how practical nursing skills are taught, learned, and maintained to strengthen competence and quality in nursing care. The inclusion of participants reflects the current composition of this network, which includes collaborating institutions from these three countries.

### 2.1 Design

A quantitative cross-sectional design was chosen (Wang and Cheng, 2020). A survey was conducted to investigate practical nursing skills practiced during clinical placement in bachelor nursing education in the Scandinavian countries.

### 2.2 Setting

When this study was conducted, the proportion of time spent in clinical placement varied among Denmark, Norway, and Sweden. Clinical practice occurs in diverse healthcare settings during education, including in medical, surgical, and orthopedic wards, mental healthcare, municipal healthcare facilities, and homes for the elderly. Although supervision during clinical placement is not strictly regulated, faculty at each educational institution, in collaboration with the nurses in the practice settings, play a crucial role in guiding students, introducing them to practical skills, and preparing them for their upcoming profession (European Commission, 2013).

### 2.3 Participants

Students enrolled in semesters one through six of bachelor nursing education programs in six Scandinavian universities or university colleges, with two institutions from each of Denmark, Norway, and Sweden, were invited to participate. Nursing students who attended clinical placement during the data collection period were invited to participate in the study, and 827 students agreed. However, 121 students were excluded due to incomplete questionnaire responses (i.e., they answered under one-third-of the questions), resulting in 706 participants. Most participants were female. For detailed participant demographics and characteristics, see Table 1.

**Table 1:** Participant demographics and characteristics.

Participants, total and per country *n* (%)	Age, years m (range)	Gender *n* (%)	Year *n* (%)
Total, 706 (100)	26 (19–62)	Female, 641 (91)	1 252 (36) 2 266 (37) 3 188 (27)
Denmark, 288 (41)	25 (19–54)	Female, 273 (95)	1 132 (46) 2 75 (26) 3 81 (28)
Norway, 240 (34)	27 (19–61)	Female, 217 (90)	1 89 (37) 2 75 (31) 3 76 (32)
Sweden, 178 (25)	27 (19–62)	Female, 151 (85)	1 31 (17) 2 116 (66) 3 31 (17)

Comprehensive data on the total number of eligible students across all semesters and sites were not consistently available; it was not possible to calculate the proportion of respondents in relation to the total population of nursing students. The sample should therefore be interpreted as a convenience sample.

### 2.4 The survey

Data were collected using a web-based questionnaire. In 2019, the RiNS members (https://www.rins.dk), all of whom had prior experience in teaching practical nursing skills at universities or university colleges as well as supervising students during their clinical placements, created a questionnaire including open-ended questions. Initially, they identified relevant practical nursing skills from six course curricula, ensuring that the content reflected educational practice across different contexts and countries. The draft questionnaire was pilot tested with both nursing students and academic staff. Their feedback led to minor revisions to improve clarity, relevance, and completeness, including the addition and refinement of items related to preceptor supervision. Two RiNS members from each Scandinavian country translated the questionnaire into their respective languages (Bölenius et al., 2024).

A specific question regarding supervision was added for this study, and the questionnaire also included questions on the frequency of performing the different skills during clinical placements. The questionnaire addressed various practical skills, categorized into seven nursing skill domains: *respiration, circulation, elimination, nutrition, activities, skin care*, and *medication* (see, Table 2). Within each domain, the questionnaire presented 4–11 examples of specific skills and the occasions/number of times they were performed under supervision. The questionnaire achieved acceptable face validity (Bölenius et al., 2024).

### 2.5 Data collection

The data collection started in 2021 and continued until 2023, as student intake varied between autumn and spring at the different institutions, and because the data collection was competing with another ongoing study. Researchers affiliated with the RiNS group were responsible for disseminating and compiling answers from each country. The head of the nursing department at each university or university college agreed to the researchers posting information about the project on the internal course websites of each educational institution. Written information about the study and its objectives was sent via e-mail, along with the survey’s web link, to the nursing students. The inclusion criterion for participation was that the students should have completed their clinical placements for the term in which the survey was distributed. One reminder e-mail was sent two weeks before the survey period ended.

### 2.6 Statistical analysis

The collected data were entered into the statistical software package . Statistical Package for the Social Sciences (SPSS) version 29 for Windows (Pallant, 2020) and analyzed using descriptive statistics. The frequency of opportunities for practical skill learning, both with and without supervision, was reported in the raw data as 0 times, 1–3 times, 4–10 times, and >10 times. The categorized responses were then subsequently dichotomized into 1 ≥ 10 = “yes, have practiced,” and 0 = “no, have not practiced at all,” for each category. For nursing students’ self-reported answers to be included in the analyses, they had to answer “yes” or “no” to all questions in each practical skill category and complete more than one-third-of the questions.

To analyze nursing students’ opportunities to perform practical nursing skills and to what extent and how frequently these skills were practiced during their clinical placements, the categorical variables were reported as absolute numbers and percentages. In the result, this is also presented in terms of the most and least frequently practiced skills.

To identify the frequency with which nursing students received guidance on practical skills from preceptors, and whether there was a specific group of practical skills in which students received more training under supervision, compared with other practical skills, we analyzed a subgroup of participants, as shown in Table 2. Participants who reported performing a practical skill (answering “yes”) formed a subgroup and were analyzed further to determine whether they had the opportunity to practice under supervision (“Yes”) or without supervision (“No”). The categorical variables are reported as absolute numbers and percentages.

The columns in the, Table 2, Table 3, present numbers and percentages for each year and the total across all three years. Values ≥70% for the proportion of skills practice (“Yes” or “No”) are highlighted in bold.

### 2.7 Ethics

This study received approval from the research ethics authorities in each participating country, i.e., the Danish Code of Conduct for Research Integrity, the Norwegian Social Science Data Service (no. 365,918), and the Swedish Ethical Review Authority (registration number. 2021–06,095-01), and upheld the principles of the Declaration of Helsinki. Participation in the study was voluntary, confidentiality was assured, and informed consent was obtained in the questionnaire from the participants. The study did not involve handling sensitive data. However, answering questions might be demanding for participants and challenge students’ knowledge (Polit and Beck, 2020). Completing the questionnaires could prompt reflections and follow-up questions, so the participants in this study were given the opportunity to ask questions after completing the questionnaire and were promised feedback on the study results. The benefits of the study were considered to outweigh any associated risks.

## 3 Results

Nursing students’ self-assessments revealed notable variations in the practical skills performed during their clinical placements in their bachelor’s nursing education. The results revealed significant variation, with some practical skills being performed by most students. In contrast, others were performed to a lesser extent or not at all. The results show that nursing students at the included institutions lacked opportunities to practice a wide range of practical skills during their clinical placements, as shown in Table 2.

**Table 2:** The numbers and percentages of practical skills practiced (yes/no) across different years reported by nursing students (>70% shown in bold).

	Year 1, n = 252		Year, 2 n = 266		Year 3, n = 188		Total, n = 706	
	Yes, *n* (%)	No, *n* (%)	Yes, *n* (%)	No, *n* (%)	Yes, *n* (%)	No, *n* (%)	Yes, *n* (%)	No, *n* (%)
**Categories and types of practical skills**								
**Respiration**								
Measuring saturation	**187 (77)**	55 (23)	**237 (90)**	27 (10)	**158 (91)**	15 (9)	**582 (86)**	97 (14)
Administering oxygen	93 (39)	148 (61)	**190 (72)**	74 (28)	113 (65)	61 (35)	365 (54)	309 (46)
Practicing respiration rate observation/assessment	**206 (84)**	38 (16)	**230 (88)**	32 (12)	**154 (89)**	19 (11)	**590 (87)**	89 (13)
Preventing secretion stagnation	62(25)	**182 (75)**	124 (47)	139 (53)	75 (43)	98 (57)	261 (38)	419 (62)
Preventing secretion stagnation with pep or CPAP	75 (31)	167 (69)	154 (59)	109 (41)	91 (53)	82 (47)	320 (48)	358 (53)
Suctioning the upper airways	14 (6)	**229 (94)**	33 (12)	**231 (87)**	39 (23)	**133 (77)**	86 (13)	**593 (88)**
Handling sputum samples	19 (8)	**222 (92)**	41 (16)	**222 (84)**	52 (30)	**121 (70)**	112 (16)	**565 (84)**
**Circulation**								
Blood pressure measurement	**231 (95)**	12 (5)	**253 (96)**	11 (4)	**165 (96)**	6 (4)	**649 (96)**	29 (4)
Pulse monitoring	**215 (89)**	26 (11)	**248 (94)**	16 (6)	**165 (96)**	6 (4)	**628 (93)**	48 (7)
Assessing and/or monitoring ECG	46 (19)	**195 (81)**	159 (60)	104 (40)	87 (51)	83 (49)	292 (43)	382 (57)
Peripheral venous catheterization, insert	35 (15)	**205 (85)**	175 (66)	89 (34)	95 (56)	76 (44)	305 (45)	370 (55)
Peripheral venous catheterization, care	69 (29)	**172 (71)**	**197 (75)**	67 (25)	109 (64)	61 (36)	375 (56)	300 (44)
Peripheral venous catheterization, removing	85 (35)	156 (65)	**211 (80)**	52 (20)	**120 (70)**	51 (30)	416 (62)	259 (38)
Central venous catheter care	32 (13)	**209 (87)**	145 (55)	119 (45)	83 (48)	88 (52)	260 (38)	416 (62)
Preparing and administering IV fluid	82 (34)	159 (66)	**206 (78)**	58 (22)	**124 (73)**	46 (27)	412 (61)	263 (39)
Preparing and administering blood transfusion	36 (15)	**204 (85)**	114 (43)	149 (57)	74 (44)	96 (56)	225 (34)	449 (67)
Measuring b-glucose	**174 (72)**	67 (28)	**226 (86)**	37 (14)	**148 (87)**	23 (13)	**548 (81)**	127 (19)
Blood sampling from different catheters (e.g., CVC)	46 (19)	**195 (81)**	132 (50)	131 (50)	46 (27)	**123 (73)**	224 (33)	449 (67)
Compression therapy for venous ulcers	123 (52)	114 (48)	158 (60)	105 (40)	99 (58)	71 (42)	380 (57)	290 (43)
Compression therapy bandage	98 (41)	141 (59)	120 (46)	143 (55)	102 (60)	69 (40)	320 (47)	353 (53)
Temperature monitoring	**194 (81)**	47 (19)	**235 (90)**	27 (10)	**153 (90)**	17 (10)	**582 (86)**	91 (14)
**Elimination**								
Providing bedpan	**186 (74)**	65 (26)	**200 (75)**	66 (25)	**142 (75)**	46 (25)	**528 (75)**	177 (25)
Urinary catheterization, female	47 (19)	**205 (81)**	69 (26)	**195 (74)**	71 (38)	117 (63)	187 (27)	**517 (73)**
Urinary catheterization, male	50 (20)	**201 (80)**	85 (32)	180 (68)	82 (44)	105 (56)	217 (31)	486 (69)
Removing urinary catheter	90 (36)	160 (64)	145 (55)	119 (45)	121 (65)	66 (35)	356 (51)	345 (49)
Applying uridome	24 (10)	**225 (90)**	24 (9)	**241 (91)**	38 (20)	**149 (80)**	86 (12)	**615 (88)**
Sampling urine	83 (34)	163 (66)	127 (48)	139 (52)	98 (53)	88 (47)	308 (44)	390 (56)
Administering enema	52 (20)	**197 (80)**	75 (28)	**191 (72)**	66 (35)	120 (65)	193 (28)	**508 (72)**
Changing ostomy pouch	107 (43)	143 (57)	111 (42)	154 (58)	102 (55)	85 (45)	320 (46)	382 (54)
Stoma care	79 (31)	172 (69)	82 (31)	**183 (70)**	83 (44)	104 (55)	244 (35)	459 (65)
**Nutrition**								
Serving food	**224 (91)**	23 (9)	**224 (84)**	42 (16)	**143 (77)**	43 (23)	**592 (85)**	108 (15)
Helping patient eat	137 (55)	112 (45)	87 (33)	177 (67)	81 (44)	104 (56)	305 (44)	393 (56)
Administering enteral nutrition (V-sond/Peg)	39 (16)	**211 (84)**	59 (22)	**207 (78)**	63 (34)	123 (66)	161 (23)	**541 (77)**
Inserting nasogastric sond	2 (1)	**246 (99)**	1 (0.5)	**265 (99.5)**	3 (2)	**182 (98)**	6 (1)	**693 (99)**
Administering parental nutrition (e.g., CVC, PVC)	7 (3)	**241 (97)**	67 (25)	**199 (75)**	40 (22)	**145 (78)**	114 (16)	**585 (84)**
**Activity, positioning, and mobilization of patients**								
Mobilization from bed to chair	**220 (87)**	32 (13)	**225 (85)**	40 (15)	**149 (79)**	39 (21)	**594 (84)**	111 (16)
Transferring patient with lift	**183 (73)**	69 (27)	136 (51)	130 (49)	109 (58)	78 (42)	428 (61)	278 (39)
Positioning patient in Fowler’s position	150 (60)	101 (40)	159 (60)	107 (40)	119 (63)	69 (37)	428 (61)	277 (39)
Positioning patient in bed	**175 (70)**	74 (30)	176 (66)	90 (34)	117 (62)	71 (38)	468 (67)	235 (33)
**Skin care**								
Making bed with patient in bed	146 (60)	98 (40)	181 (68)	84 (32)	113 (63)	66 (37)	440 (64)	248 (36)
Assisting with shower	**214 (88)**	30 (12)	146 (55)	119 (45)	124 (69)	55 (31)	**484 (70)**	204 (30)
Toothbrushing	170 (69)	76 (31)	120 (45)	144 (55)	117 (65)	62 (35)	407 (59)	282 (41)
Oral hygiene	113 (46)	131 (54)	118 (44)	147 (56)	80 (45)	89 (55)	311 (45)	377 (55)
Personal hygiene	**216 (89)**	27 (11)	**206 (78)**	59 (22)	**137 (76)**	42 (24)	**559 (81)**	128 (19)
Surgical wound care	91 (37)	153 (63)	155 (58)	110 (42)	121 (68)	58 (32)	367 (53)	321 (47)
Chronic wound care	114 (47)	130 (53)	134 (51)	131 (49)	121 (69)	55 (31)	369 (54)	316 (46)
Removing stiches or staples	75 (31)	169 (69)	115 (43)	150 (57)	106 (59)	73 (41)	296 (43)	392 (57)
Handling drain	44 (18)	**200 (82)**	122 (46)	143 (54)	87 (49)	91 (51)	253 (37)	434 (63)
Post-mortem care	44 (19)	**190 (81)**	50 (19)	**210 (80)**	56 (34)	110 (66)	150 (23)	**510 (77)**
**Medication**								
Administering epidural analgesia	16 (7)	**223 (93)**	51 (19)	**211 (81)**	34 (20)	**133 (80)**	101 (15)	**567 (85)**
Care of epidural catheter	15 (6)	**222 (94)**	49 (19)	**212 (81)**	33 (20)	**135 (80)**	97 (15)	**569 (85)**
Handling pills	158 (66)	81 (34)	**249 (95)**	12 (5)	**159 (94)**	10 (6)	**566 (85)**	103 (15)
Rectal administration	70 (29)	170 (71)	83 (32)	178 (68)	93 (55)	76 (45)	246 (37)	424 (63)
Administering medication via feeding tube	47 (20)	**190 (80)**	86 (33)	175 (67)	85 (51)	83 (49)	218 (33)	448 (67)
Administering infusions	67 (28)	**169 (72)**	**208 (80)**	53 (20)	113 (68)	52 (32)	388 (59)	274 (41)
Administering IM injections	55 (23)	**182 (77)**	79 (30)	**181 (70)**	96 (57)	71 (43)	230 (35)	434 (65)
Administering SC injections	116 (49)	122 (51)	**216 (83)**	45 (17)	**136 (82)**	30 (18)	**468 (70)**	197 (30)
Handling infusion pump	49 (21)	**188 (79)**	152 (58)	109 (42)	99 (69)	67 (40)	300 (45)	364 (55)
Applying cream	134 (56)	104 (44)	131 (50)	130 (50)	**117 (70)**	50 (30)	382 (57)	284 (43)
Administering inhalations	105 (45)	130 (55)	185 (62)	96 (37)	115 (69)	52 (31)	385 (58)	278 (42)

**Table 3:** The numbers and percentages of practical skills practiced under supervision (yes/no) and differences between years of undergraduate education and total reported by faculties in Scandinavian countries (>70% shown in bold).

	Year 1		Year 2		Year 3		Total	
	Yes, *n* (%)	No, *n* (%)	Yes, *n* (%)	No, *n* (%)	Yes, *n* (%)	No, *n* (%)	Yes, *n* (%)	No, *n* (%)
**Categories and types of practical skills**								
**Respiration**								
Measuring saturation	**133 (71)**	54 (29)	130 (55)	107 (45)	65 (41)	93 (59)	328 (56)	254 (44)
Administering oxygen	**81 (88)**	11 (12)	**172 (91)**	18 (9)	**105 (93)**	8 (7)	**358 (91)**	37 (9)
Practicing respiration rate observation/assessment	128 (63)	76 (37)	115 (50)	115 (50)	58 (38)	96 (62)	47 (39)	74 (61)
Preventing secretion stagnation	**44 (71)**	18 (29)	**102 (82)**	22 (18)	**60 (80)**	15 (20)	**206 (79)**	55 (21)
Preventing secretion stagnation with pep or CPAP	**55 (73)**	20 (27)	**115 (75)**	39 (25)	**73 (80)**	18 (20)	**243 (76)**	77 (24)
Suctioning the upper airways	**14(100)**	0 (0)	**32 (97)**	1 (3)	**39 (100)**	0 (0)	**85 (99)**	1 (1)
Handling sputum samples	**15 (79)**	4 (21)	**29 (71)**	12 (29)	**42 (82)**	9 (18)	**86 (77)**	25 (23)
**Circulation**								
Blood pressure measurement	**168 (73)**	61 (27)	137 (54)	116 (46)	83 (51)	80 (49)	388 (60)	257 (40)
Pulse monitoring	140 (65)	74 (35)	121 (49)	127 (51)	70 (42)	95 (58)	331 (53)	296 (47)
Assessing and/or monitoring ECG	**44 (96)**	2 (4)	**135 (85)**	24 (15)	**76 (87)**	11 (13)	**255 (87)**	37 (13)
Peripheral venous catheterization, insert	**32 (91)**	3 (9)	**164 (94)**	11 (6)	**88 (93)**	7 (7)	**284 (93)**	21 (7)
Peripheral venous catheterization, care	**61 (88)**	8 (12)	**171 (87)**	26 (13)	**85 (79)**	23 (21)	**317 (85)**	57 (15)
Peripheral venous catheterization, removing	**71 (84)**	13 (16)	139 (66)	72 (34)	73 (61)	47 (39)	283 (68)	132 (32)
Central venous catheter care	**31 (97)**	1 (3)	**139 (96)**	6 (4)	**79 (95)**	4 (5)	**249 (96)**	11 (4)
Preparing and administering IV fluid	**77 (94)**	5 (6)	**297 (97)**	6 (3)	**113 (91)**	11 (9)	**390 (95)**	22 (5)
Preparing and administering blood transfusion	**35 (97)**	1 (3)	**114 (100)**	0 (0)	**75 (100)**	0 (0)	**224 (99)**	1 (1)
Measuring b-glucose	**143 (83)**	30 (17)	140 (62)	86 (38)	90 (61)	57 (39)	373 (68)	173 (32)
Blood sampling from different catheters (e.g., CVC)	**44 (96)**	2 (4)	**110 (83)**	22 (17)	**34 (74)**	12 (26)	**188 (84)**	36 (16)
Compression therapy for venous ulcers	68 (56)	54 (44)	73 (46)	84 (54)	57 (58)	42 (42)	198 (52)	180 (48)
Compression therapy bandage	**83 (86)**	14 (14)	**89 (74)**	31 (26)	**86 (85)**	15 (15)	**258 (81)**	60 (19)
Temperature monitoring	123 (64)	68 (36)	113 (48)	121 (52)	68 (45)	84 (55)	304 (53)	273 (47)
**Elimination**								
Providing bedpan	97 (52)	89(48)	110 (55)	90 (45)	78 (55)	64 (55)	285 (54)	243 (46)
Urinary catheterization, female	**45 (96)**	2(4)	**52 (75)**	17 (25)	**64 (90)**	7 (10)	**161 (86)**	26 (14)
Urinary catheterization, male	**48 (96)**	2(4)	**68 (81)**	16 (19)	**76 (93)**	6 (7)	**192 (89)**	24 (11)
Removing urinary catheter	**80 (89)**	10 (11)	**109 (76)**	35 (24)	**88 (73)**	33 (27)	**277 (78)**	78 (22)
Applying uridome	**19 (79)**	5 (21)	16 (67)	8 (33)	22 (58)	16 (42)	57 (66)	29 (34)
Sampling urine	**64 (77)**	19 **(**23)	**94 (74)**	33 (26)	60 (61)	38 (39)	**218 (71)**	90 (29)
Administering enema	**43 (83)**	9 (17)	**54 (72)**	21 (28)	44 (67)	22 (33)	**141 (73)**	52 (27)
Changing ostomy pouch	**93 (87)**	14(13)	**85 (77)**	26 (23)	76 (75)	26 (25)	**254 (79)**	66 (21)
Stoma care	**70 (89)**	9 (11)	**68 (83)**	14 (17)	67 (82)	15 (18)	**205 (84)**	38 (16)
**Nutrition**								
Serving food	43 (19)	**181 (81)**	20 (9)	**204 (91)**	17 (12)	**125 (88)**	80 (14)	**510 (86)**
Helping patient eat	6 (4)	**130 (96)**	8 (9)	**79 (91)**	7 (9)	**74 (91)**	21 (7)	**283 (93)**
Administering enteral nutrition (V-sond/Peg)	12 (31)	27 (69)	24 (41)	35 (59)	19 (30)	**44 (70)**	55 (34)	106 (66)
Inserting nasogastric sond	0 (0)	**2 (100)**	**1 (100)**	0 (0)	0 (0)	**3 (100)**	1 (17)	**5 (83)**
Administering parental nutrition (e.g., CVC, PVC)	2 (29)	5 (71)	33 (49)	34 (51)	17 (44)	22 (56)	52 (46)	61 (54)
**Activity, positioning, and mobilization of patients**								
Mobilization from bed to chair	**165 (75)**	55 (25)	**160 (71)**	65 (29)	**103 (70)**	45 (30)	**428 (72)**	165 (28)
Transferring patient with lift	**139 (76)**	44 (24)	**113 (83)**	23 (17)	**92 (85)**	16 (15)	**344 (81)**	83 (19)
Positioning patient in Fowler’s position	**112 (76)**	36 (24)	**125 (79)**	34 (21)	**83 (70)**	36 (30)	**320 (75)**	106 (25)
Positioning patient in bed	**128 (73)**	47 (27)	**132 (75)**	44 (25)	**91 (78)**	26 (22)	**351 (75)**	117 (25)
**Skin care**								
Making bed with patient in bed	95 (66)	48 (34)	128 (71)	53 (29)	88 (79)	24 (21)	311 (71)	125 (29)
Assisting with shower	97 (46)	112 (54)	46 (32)	99 (68)	48 (39)	76 (61)	191 (40)	287 (60)
Toothbrushing	97 (58)	71 (42)	34 (28)	86 (72)	43 (37)	73 (63)	174 (43)	230 (57)
Oral hygiene	65 (58)	47 (42)	68 (58)	50 (42)	44 (55)	36 (45)	177 (57)	133 (43)
Personal hygiene	**157 (73)**	58 (27)	121 (59)	85 (41)	87 (63)	50 (37)	365 (65)	193 (35)
Surgical wound care	**89 (98)**	2 (2)	**136 (88)**	19(12)	**113 (93)**	8 (7)	**338 (92)**	29 (8)
Chronic wound care	**109 (96)**	4 (4)	**125 (93)**	9 (7)	**115 (95)**	6 (5)	**349 (95)**	19 (5)
Removing stiches or staples	**68 (91)**	7 (9)	**102 (89)**	13 (11)	**93 (88)**	13 (12)	**263 (89)**	33 (11)
Handling drain	**42 (96)**	2 (4)	**114 (93)**	8 (7)	**86 (99)**	1 (1)	**242 (96)**	11 (4)
Post-mortem care	**33 (85)**	6 (15)	**46 (92)**	4 (8)	**53 (95)**	3 (5)	**137 (91)**	13 (9)
**Medication**								
Administering epidural analgesia	**15 (94)**	1 (7)	**49 (96)**	2 (4)	**32 (94)**	2 (6)	**96 (95)**	5 (5)
Care of epidural catheter	**15 (100)**	0 (0)	**49 (100)**	0 (0)	**33 (100)**	0 (0)	**97 (100)**	0 (0)
Handling pills	**145 (93)**	11 (7)	**235 (94)**	14 (6)	**151 (95)**	8 (5)	**531 (94)**	33 (6)
Rectal administration	**56 (80)**	14 (20)	**64 (77)**	19 (23)	**67 (73)**	25 (27)	**187 (76)**	58 (24)
Administering medication via feeding tube	**43 (91)**	4 (4)	**84 (98)**	2 (2)	**79 (93)**	6 (7)	**206 (94)**	12 (6)
Administering infusions	**64 (95)**	3 (5)	**205 (99)**	3 (1)	**111 (98)**	2 (2)	**380 (98)**	8 (2)
Administering IM injections	**52 (96)**	2 (4)	**78 (99)**	1 (1)	**93 (97)**	3 (3)	**223 (97)**	6 (3)
Administering SC injections	**110 (96)**	5 (4)	**191 (89)**	24 (11)	**124 (91)**	12 (9)	**425 (91)**	41 (9)
Handling infusion pump	**47 (96)**	2 (4)	**148 (97)**	4 (3)	**96 (97)**	3 (3)	**291 (97)**	9 (3)
Applying cream	**96 (74)**	34 (26)	**96 (73)**	35 (27)	**85 (73)**	31 (27)	**277 (73)**	100(27)
Administering inhalations	**75 (72)**	29 (28)	**133 (81)**	32 (19)	**86 (75)**	29 (25)	**294 (77)**	90 (23)

There was considerable consistency in practicing different nursing skills across the years, indicating that some of the practical skills were not being performed during clinical placements. The results show that a preceptor most often facilitated nursing students who had the opportunity to perform practical skills, although notable gaps in supervision were observed in some specific skills (Table 3).

### 3.1 Respiration

In the *respiratory care* category, basic assessment skills, such as monitoring oxygen levels and respiratory rates, were among the most frequently performed practical skills (see Table 2). Across all study years, measuring oxygen levels and assessing respiratory rates were the most commonly performed skills, receiving performance scores of 86% (*n* = 582) and 87% (*n* = 590), respectively. In contrast, more advanced procedures, including suctioning the upper airways and handling sputum samples, were performed less frequently, resulting in 88% (*n* = 593) and 84% (*n* = 565), respectively, of the students never having performed these skills. Practicing respiratory skills under supervision (see Table 3) was relatively common (overall mean 74%), particularly for suctioning the upper airways (*n* = 85, 99%) and administering oxygen (*n* = 358, 91%). Notably, a smaller proportion of students (*n* = 77, 24%) reported performing advanced interventions, such as preventing secretion stagnation with Positive Expiratory Pressure (PEP) or Continuous Positive Airway pressure (CPAP), without supervision.

### 3.2 Circulation

In the *circulation-related skills* category, basic measurements such as blood pressure, pulse, and temperature were consistently performed throughout the nursing education. These were also the most frequently performed skills overall and in each specific year: blood pressure measurement (*n* = 649, 96%), pulse monitoring (*n* = 628, 93%), blood glucose measurement (*n* = 548, 81%), and temperature monitoring (*n* = 582, 86%). In contrast, opportunities to engage in more advanced procedures, such as preparing and administering blood transfusions or handling central venous catheters, were limited, and most students did not perform these tasks. Notably, >50% of students lacked the opportunity to insert, handle, or remove peripheral venous catheters. When advanced practical nursing skills were performed, they were typically carried out under supervision, whereas basic tasks such as blood pressure measurement were more often performed independently by 40%of the students.

### 3.3 Elimination

In the *elimination* category, fundamental tasks such as assisting with toileting aids were more frequently performed than invasive procedures. The most frequently performed practical nursing skill overall, and consistently across all years, was providing a bedpan (*n* = 528, 75%). In contrast, more advanced invasive procedures, such as urinary catheterization for females (*n* = 517, 73%) and administering enemas (*n* = 508, 72%), were relatively rare, particularly during the first two years. Applying a uridome was the least frequently performed skill both annually and in total (*n* = 615, 88%). Opportunities for supervision in this category appeared to decrease slightly in later years, reflecting a general trend towards reduced supervised practice across several categories within elimination-related tasks.

### 3.4 Nutrition

In the *nutrition* category, the most frequently performed practical nursing skill overall was serving food (*n* = 592, 85%). In contrast, the least performed skills were administering enteral nutrition (nasogastric tube/PEG) (*n* = 541, 77%), inserting a nasogastric tube (*n* = 693, 99%), and administering parenteral nutrition (PVC, CVC, etc.) (*n* = 585, 84%). These results were consistent when analyzed year by year, except for the administration of enteral nutrition in the third year. Serving food and assisting patients with eating were performed without supervision both annually and in total. Notably, there were very few opportunities to practice inserting a nasogastric tube, and in 83% of those instances, students (*n* = 5) reported practicing without supervision.

### 3.5 Patient activity and mobilization

In the categories of *patient activity and mobilization*, transferring patients, for example from bed to chair, emerged as the most frequently practiced. However, these tasks were generally not performed without oversight, highlighting the high presence of supervision throughout. The most commonly performed nursing practical skill was transferring patients from bed to chair (*n* = 594, 84%). This result was consistent when analyzed year by year. However, none of the skills in this category exceeded 70% in terms of opportunities for practice without supervision. All those who performed these activities, both year by year and in total, did so under supervision.

### 3.6 Skin care

In the *skin care* category, routine hygiene tasks were regularly performed, with the most frequently performed practical nursing skills being assisting during showers (*n* = 484, 70%) and providing personal hygiene (*n* = 559, 81%). In contrast, more specialized or sensitive procedures, such as wound care, removing stitches and staples, handling drains, and post-mortem care, were less frequently performed and typically conducted under supervision. Post-mortem care was the least performed skill overall and in the first two years (*n* = 510, 77%). While personal hygiene was supervised mainly in the first year, advanced tasks consistently required supervision across all years. Notably, post-mortem care was performed without supervision in 9% of cases.

### 3.7 Medication

Within the category of *medication administration*, students gained the most experience with routine practices such as handling oral medications and administering subcutaneous injections. These were also the most frequently performed practical nursing skills in years two and three and overall (*n* = 566, 85% and *n* = 468, 70%, respectively). In contrast, more specialized procedures, including epidural administration and managing epidural catheters, were among the least frequently performed skills (*n* = 567, 85% and *n* = 569, 85%), with these patterns remaining consistent across all three years. Opportunities to administer intramuscular injections were also limited, as 70% of students in the second year reported not performing this skill, and several medication-related tasks were not undertaken at all during the first year. Overall, medication procedures were predominantly performed under supervision, with each category exceeding 70%, indicating restricted opportunities for independent practice.

See the, Table 2, Table 3, for a more detailed account of each category.

## 4 Discussion

The overall aim of this study was to explore nursing students’ opportunities to perform practical skills during clinical placements, and the degree to which they were supervised by preceptors. There was considerable variation in the types of practical skills performed, with some skills being frequently practiced and others rarely or not at all. One interpretation is that this variation may limit nursing students’ opportunities to perform practical skills during clinical placements, and that they may therefore lack basic knowledge of practical nursing skills, in turn affecting the quality and safety of patient care. Furthermore, preceptors most often facilitated nursing students who had the opportunity to perform practical skills. As mentioned in the introduction, learning in clinical placements accounts for almost half of nursing education (European Commission, 2013; Henriksen et al., 2020), placing high demands on the quality of this component. Our results show that some essential practical skills are never practiced by certain students during their clinical placement. For example, >50% of students reportedly lacked the opportunity to insert, handle, and remove peripheral venous catheters. The consistent results across study years regarding the performance of certain nursing skills suggest that some practical skills are systematically overlooked during clinical placements, potentially leading to missed learning opportunities and unachieved learning outcomes throughout the nursing education. This lack of opportunities to practice essential procedures ultimately poses challenges for newly graduated nurses, who may struggle to master the insertion of peripheral venous catheters when they are not permitted to perform the procedure during their training (Ravik et al., 2023). However, in the present study, >80% of the students frequently practiced blood pressure measurement and pulse monitoring. Similarly, Bölenius et al. (2024) found that these skills were also commonly practiced at simulation centers.

One suggestion is that the practical nursing skills commonly practiced both at simulation centers and during clinical placements could be prioritized for clinical placements, allowing simulation centers to focus on training in other, less frequently practiced skills. This pattern of overlap between skills practiced in simulation centres and clinical placements suggests that more structured guidance and follow-up may be beneficial in clarifying which practical skills should be prioritized during clinical placements and in nursing education. Cant et al. (2021) support our interpretation of the results, noting that the learning and structure during the students’ clinical practice are of utmost importance and that the structure surrounding their practical skills learning is crucial for their professional development. Students require clear expectations and continuous feedback to understand what is expected of them throughout their education. The absence of these elements may negatively impact their learning outcomes.

In this study, a substantial number of practical skills performed during clinical placement appeared to be practiced without the presence of a preceptor. This lack of supervision may contribute to inconsistencies in skill acquisition, hinder deeper learning, and ultimately have a negative impact on patient safety. Since there is no consensus on the learning process for practical skills in nursing education, due to diverse learning modalities and assessment tools, there is a risk that the students’ practical competence may be insufficient (Hilleren et al., 2022; Reierson et al., 2024). Our findings show that while preceptors generally facilitated skill practice, notable gaps in supervision were observed for specific procedures such as the administration of enteral nutrition or assisting with oral hygiene. Addressing these gaps is crucial for preparing competent and confident future nurses. A qualitative study found that nurse educators wanted to prepare a learning plan before clinical education, allowing for person-centered learning that meets students’ prerequisites and expectations (Nyqvist et al., 2020). This type of learning plan might help prioritize particular practical skills to focus on during clinical placements. Prashar et al. (2022) suggested three ways for students to maximize exposure to practical skills both within and outside the clinical setting: making the most of existing opportunities for learning; creating new opportunities for learning; and supporting reflective practice. The lack of opportunities for nursing students to practice practical nursing skills under preceptor supervision during clinical placements may affect learning, as guidance from experienced nurses is essential for developing clinical competence. Mastering practical skills often involves multiple steps and requires deep, comprehensive learning (Nielsen et al., 2013). However, supervision in clinical settings frequently focuses on informing, reminding, and correcting students, rather than on fostering reflection and dialogue. This approach risks promoting superficial learning, which may be insufficient for achieving proficiency in complex practical skills (Ravik and Bjørk, 2022).

The present study results showed that nursing students were often supervised when practicing skills in medication administration. However, some skills related to nutrition, such as assisting patients with eating, were often practiced without supervision. This lack of supervision may jeopardize the learning of those skills and, in later stages, perhaps even prompt consideration regarding risks to patient safety. One interpretation is that medication administration is suggested to be a high-risk practical skill and that assisting in eating is given undue importance. However, insufficient training and supervision in commonly performed practical nursing skills, such as oral care, can, in later stages, lead to serious patient safety risks. Good oral health has been shown to prevent hospital-acquired pneumonia (Munro et al., 2022) and improve quality of life and safety for patients with chronic conditions (Petropoulou et al., 2024) and intubated patients (Winning et al., 2021). Preventing hospital-acquired pneumonia is a critical yet often neglected aspect of practical oral care (Munro et al., 2022). Poor-quality and infrequent oral care are key barriers to prevention and cause significant mortality and healthcare costs (Winning et al., 2021). A shift in nursing perspectives is needed to ensure that commonly performed practical skills are not undervalued, but instead recognized as essential for student learning and reflection. Preceptors must also acknowledge the importance of practical skills as a key aspect of patient safety and clinical competence.

Earlier studies emphasize that clinical supervision is a component of nursing education, particularly in the development of practical skills, but also to foster professional identity and ensure patient safety (Vabo et al., 2022). Ravik and Bjørk (2022) found that supervision by RNs during practical skill training such as peripheral vein cannulation plays a critical role in enabling students to perform complex procedures safely and effectively. Similarly, Amsrud et al. (2015) showed that clinical supervision strengthens nursing students’ interpersonal, professional, and communication skills, all of which are essential for the safe and competent performance of practical procedures. These authors also highlighted that supervision contributes not only to individual competence but also to the broader safety culture within clinical settings. Supporting this, Zaitoun et al. (2023) demonstrated a positive correlation between nurses’ clinical competence and patient safety culture, underscoring the importance of implementing structured supervision that facilitates skill development (Tuomikoski et al., 2020). In line with these previous findings, our study further illustrates considerable variation in nursing students’ opportunities to practice practical skills during clinical placements, particularly regarding whether such practice occurs under supervision. This is also highlighted in a recently published study emphasizing the need to examining the effects of various interventions aimed at improving nursing students’ learning outcomes (Hitzek et al., 2026).

Despite the importance of supervision, studies indicate that nursing students often have limited access to preceptors during clinical placements. As previously mentioned, a notable gap identified in our study was the limited opportunity for preceptor supervision during certain skill practices. Pitkänen et al. (2018) found that third-year nursing students reported low satisfaction with their clinical learning environment and supervision, highlighting the need for improved supervisory structures and pedagogical support during placements. In the long term, insufficient supervision can compromise patient safety. Bakker et al. (2019) reported that one cause of late dropout from nursing education was losing one’s grip on the learning process due to a lack of coaching and support. Taken together, these findings emphasize the need for clearer guidelines, structured followup, and an intentional approach to ensuring that all students are afforded equitable opportunities to practice and receive supervision across a broad range of nursing skills.

### 4.1 Strengths and limitations

A key strength of this study is its cross-sectional design, which enabled the inclusion of geographically diverse sample of nursing students across multiple semesters and institutions in three Scandinavian countries. This design is particularly suitable for capturing a broad overview of current experiences and perceptions at a specific point in time. It also allows for comparisons across different educational contexts and stages of training, thereby providing insights into potential variations between student groups. Furthermore, the cross-sectional approach facilitated efficient data collection within a limited timeframe, making it possible to explore patterns and associations in a relatively large sample. Taken together, this enhances the descriptive value of the study and supports the identification of areas for further research and educational development.

However, some limitations need to be acknowledged, as the structure and content of educational programs vary between countries, which may affect the comparability of the present results. Participants were enrolled in all semesters of the bachelor’s nursing education program. The study relied on a convenience sample, which may limit the generalizability of the findings. Additionally, the questionnaire used was long and comprehensive, which likely contributed to participant dropout, since some students did not complete the full survey. Furthermore, a limitation of the study is that the response rate for each included nursing programme could not be calculated, as data collection was conducted during an academic term when student enrolment fluctuated over time. Additionally, data were collected at different time points across the nursing programmes and across the three countries.

Another limitation is the lack of detailed contextual data. For example, it would have been valuable to know whether students who performed the different skills with a preceptor did so alone or together with other professional groups, such as enrolled nurses. This information could have enabled a more nuanced analysis of the responses.

Finally, data collection took place during the COVID-19 pandemic, which likely contributed to the high dropout rate and may have influenced students’ experiences and responses in ways that are difficult to control for.

## 5 Conclusion

Considerable variation was detected in the nursing students’ opportunities to perform practical nursing skills during clinical placements, which may have resulted in missed opportunities to learn essential competencies needed in their future professional roles. There is no clear consensus on how to learn these skills or on the minimum amount of time/number of opportunities needed for their practice. The findings underscore the need for clearer guidelines and structured follow-up to ensure that students consistently practice core nursing skills throughout their clinical training.

Our study identified a lack of opportunities for nursing students to learn practical skills under the supervision of a preceptor, which may affect the learning process, as guidance from experienced nurses is essential for developing clinical competence during clinical placements. Increased specialization in healthcare means that the opportunity to practice during clinical practice must be frequent, cover a wide range of skills, and incorporate adequate supervision. Moreover, a new directive from the European Union mandates that 50% of nursing education should be dedicated to clinical practice.

Identifying gaps in students’ learning experiences during clinical placements can inform improvements in nursing education and support the development of competent, confident healthcare professionals. Addressing these gaps is crucial for preparing competent and confident future nurses.

## Statement

During the preparation of this work, the author(s) used CoPilot in order to perform linguistic correction of already existing written text. In addition, the manuscript has been reviewed by an external language editing service. After using this tool/service, the author(s) reviewed and edited the content as needed and take(s) full responsibility for the content of the published article.

## Webpage research group

Research in Nursing Skills (RiNS)—Scandinavian research group (http://www.rins.dk).

## CRediT authorship contribution statement

**K Jonsson:** Writing – review & editing, Writing – original draft, Visualization, Validation, Methodology, Investigation, Formal analysis, Data curation, Conceptualization. **K Falk-Brynhildsen:** Writing – review & editing, Writing – original draft, Visualization, Validation, Methodology, Investigation, Formal analysis, Data curation, Conceptualization. **M Ravik:** Writing – review & editing, Writing – original draft, Validation, Methodology, Investigation, Data curation, Conceptualization. **IT Björk:** Writing – review & editing, Validation, Methodology, Investigation, Conceptualization. **K Blomberg:** Writing – review & editing, Validation, Methodology, Investigation, Conceptualization. **SE Husebø:** Writing – review & editing, Validation, Methodology, Investigation, Data curation, Conceptualization. **IÅ Reierson:** Writing – review & editing, Validation, Methodology, Investigation, Conceptualization. **I Sommer:** Validation, Investigation, Conceptualization. **K Bölenius:** Writing – review & editing, Writing – original draft, Visualization, Validation, Methodology, Investigation, Formal analysis, Data curation, Conceptualization.

## Declaration of competing interest

The authors declare that they have no known competing financial interests or personal relationships that could have appeared to influence the work reported in this paper.

## References

[bib0001] Amsrud K.E., Lyberg A., Severinsson E. (2015). The influence of clinical supervision and its potential for enhancing patient safety: undergraduate nursing students’ views. J. Nurs. Educ. Pr..

[bib0002] Axelsson M.T., Årestedt L., Swahnberg K., Oscarsson M. (2021). Teaching practical topics to nursing students at clinical skills centres: a total mapping of Swedish universities. Nord. J. Nurs. Res..

[bib0003] Bakker E.J., Verhaegh K.J., Kox J.H., van der Beek A.J., Boot C.R., Roelofs P.D., Francke A.L. (2019). Late dropout from nursing education: an interview study of nursing students’ experiences and reasons. Nurse Educ. Pr..

[bib0004] Bölenius K., Vesterager Stenholt B., Bjørk I.T., Reierson I.Å., Blomberg K., Husebø S.E., Ravik M. (2025). Practical skills taught in Scandinavian nursing education simulation centres: a cross-sectional survey. Scand. J. Educ. Res..

[bib0005] Cant R., Ryan C., Hughes L., Luders E., Cooper S. (2021). What helps, what hinders? Undergraduate nursing students’ perceptions of clinical placements based on a thematic synthesis of literature. SAGE Open Nurs..

[bib0006] Daneshfar M., Moonaghi H.K. (2025). The impact of clinical simulation on bridging the theory–practice gap in nursing education: a systematic review. BMC. Med. Educ..

[bib0007] EU Directive. Directive 2013/55/EU of the european parliament and of the Council of 20 November 2013 amending Directive 2005/26/EC on the recognition of professional qualifications and Regulations (EU) Directive, 2005/36 - EN - EUR-Lex, Available 260225.

[bib0008] Ewertsson M., Gustafsson M., Blomberg K., Holmstrom I.K., Allvin R. (2015). Use of technical skills and medical devices among new registered nurses: a questionnaire study. Nurse Educ. Today.

[bib0009] Hayden J.K., Smiley R.A., Alexander M., Kardong-Edgren S., Jeffries P.R. (2014). The NCSBN national simulation study: a longitudinal, randomized, controlled study replacing clinical hours with simulation in prelicensure nursing education. J. Nurs. Regul..

[bib0010] Henriksen J., Löfmark A., Wallinvirta E., Gunnarsdóttir Þ.J., Slettebø Å. (2020). European Union directives and clinical practice in nursing education in the Nordic countries. Nord. J. Nurs. Res..

[bib0011] Hilleren I.H.S., Christiansen B., Bjørk I.T. (2022). Learning practical nursing skills in simulation centers: a narrative review. Int. J. Nurs. Stud. Adv..

[bib0012] Hitzek J., Willis E., Zilezinski M., Petersen S.C., Kottner J. (2026). Learning in clinical practice: factors, outcomes, and challenges in clinical nursing education–A scoping review. J. Prof. Nurs..

[bib0013] Houghton C.E., Casey D., Shaw D., Murphy K. (2013). Students’ experiences of implementing clinical skills in the real world of practice. J. Clin. Nurs..

[bib0014] Jonsén E., Melender H.-L., Hilli Y. (2013). Finnish and Swedish nursing students’ experiences of their first clinical practice placement: a qualitative study. Nurse Educ. Today.

[bib0015] Leonardsen A.-C., Brynhildsen S., Hansen M.T., Grøndahl V.A. (2021). Supervising students in a complex nursing practice: a focus group study in Norway. BMC. Nurs..

[bib0016] Loewen P., Legal M., Gamble A., Shah K., Tkachuk S., Zed P. (2017). Learner:preceptor ratios for practice-based learning across health disciplines: a systematic review. Med. Educ..

[bib0017] Mathisen C., Heyn L.G., Jacobsen T.-I., Bjørk I.T., Hansen E.H. (2022). The use of practice education facilitators to strengthen the clinical learning environment for nursing students: a realist review. Int. J. Nurs. Stud..

[bib0018] Mathisen C., Bjørk I.T., Heyn L.G., Jacobsen T.-I., Hansen E.H. (2023). Practice education facilitators perceptions and experiences of their role in the clinical learning environment for nursing students: a qualitative study. BMC. Nurs..

[bib0019] Munro S., Phillips T., Hasselbeck R., Lucatorto M.A., Hehr A., Ochylski S. (2022). Implementing oral care as a nursing intervention to reduce hospital-acquired pneumonia across the United States Department of Veterans Affairs Healthcare System. CIN: Comput. Inform. Nurs..

[bib0020] Nielsen C., Sommer I., Larsen K., Bjørk I.T. (2013). Model of practical skill performance as an instrument for supervision and formative assessment. Nurse Educ Pr..

[bib0021] Nyqvist J., Brolin K., Nilsson T., Lindström V. (2020). The learning environment and supportive supervision promote learning and are based on the relationship between students and supervisors: a qualitative study. Nurse Educ Pr..

[bib0022] Pallant J. (2020).

[bib0023] Petropoulou P., Kalemikerakis I., Dokoutsidou E., Evangelou E., Konstantinidis T., Govina O. (2024). Oral health education in patients with diabetes: a systematic review. Healthcare.

[bib0024] Pitkänen S., Kääriäinen M., Oikarainen A., Tuomikoski A.-M., Elo S., Ruotsalainen H., Saarikoski M., Kärsämänoja T., Mikkonen K. (2018). Healthcare students' evaluation of the clinical learning environment and supervision: a cross-sectional study. Nurse Educ. Today.

[bib0025] Polit D., Beck C. (2020).

[bib0026] Pramila-Savukoski S., Juntunen J., Tuomikoski A.M., Kääriäinen M., Tomietto M., Kaučič B.M., Filej B., Riklikiene O., Vizcaya-Moreno M.F., Perez-Cañaveras R.M. (2020). Mentors' self-assessed competence in mentoring nursing students in clinical practice: a systematic review of quantitative studies. J. Clin. Nurs..

[bib0027] Prashar J., Ranasinghe C., Rao C.B. (2022). Twelve tips for medical students to enhance clinical skills learning during disrupted placements. Med. Teach..

[bib0028] Ravik M., Bjerkelund G.M., Hvalvik S., Reierson I.Å. (2025). Student nurses' learning of practical skills in hospital placements: perspectives of registered nurse mentors. Nurse Educ Pr..

[bib0029] Ravik M., Nilsen E.R., Wighus M., Mofossbakke R., Haanes G.G. (2023). Clinical placement studies during the coronavirus disease 2019 pandemic shapes new nurses: a qualitative study. Int. J. Nurs. Stud. Adv..

[bib0030] Ravik M., Bjørk I.T. (2022). Registered nurses’ supervision of nursing students during practical skill learning: a qualitative exploratory study. Scand. J. Educ. Res..

[bib0031] Ravik M., Bjørk I.T. (2023). Influence of simulation and clinical settings on peripheral vein cannulation skill learning in nursing education: a qualitative study. Int. J. Nurs. Stud. Adv..

[bib0032] Ravik M., Havnes A., Bjørk I.T. (2014). Exploring nursing students’ transfer of peripheral venous cannulation from skills centre to the clinical setting. J. Nurs. Educ. Pr..

[bib0033] Ravik M., Havnes A., Bjørk I.T. (2017). Defining and comparing learning actions in two simulation modalities: students training on a latex arm and each other’s arms. J. Clin. Nurs..

[bib0034] Reierson I.Å., Ravik M., Blomberg K., Bjørk I.T., Bölenius K., Vesterager Stenholt B., Husebø S.E. (2024). Comparing didactic approaches for practical skills learning in Scandinavian nursing simulation centres: a qualitative comparative study. J. Adv. Nurs..

[bib0035] Rudland J., Ash J., Barclay L., Bloomfield J., Bogossian F., Byrne K., Butler M., Chur-Hansen A., Clark S., Edgar A. (2025). Rethinking clinical placements: a response to changing healthcare demands. Focus Health Prof. Educ.: Multi-Prof. J..

[bib0036] Schroers G., Shrikanth S., Pfieffer J. (2023). Undergraduate nursing student experiences in American clinical learning environments: a descriptive study. Nurse Educ. Today.

[bib0037] Song Y., McCreary L.L. (2020). New graduate nurses’ self-assessed competencies: an integrative review. Nurse Educ. Pr..

[bib0038] Strobl A., Nestler N., Gnass I. (2022). Tasks and roles of newly bachelor graduates of nursing in acute care settings: a scoping review. Pflege.

[bib0039] Södersved Källestedt M.L., Asp M., Letterstål A., Widarsson M. (2020). Perceptions of managers regarding prerequisites for the development of professional competence of newly graduated nurses: a qualitative study. J. Clin. Nurs..

[bib0040] Tuomikoski A.-M., Ruotsalainen H., Mikkonen K., Kääriäinen M. (2020). Nurses’ experiences of their competence at mentoring nursing students during clinical practice: a systematic review of qualitative studies. Nurse Educ. Today.

[bib0041] Vabo G., Slettebø Å., Fossum M. (2022). Nursing students’ professional identity development: an integrative review. Nord. J. Nurs. Res..

[bib0042] Waldner M.H., Olson J.K. (2007). Taking the patient to the classroom: applying theoretical frameworks to simulation in nursing education. Int. J. Nurs. Educ. Sch..

[bib0043] Wang X., Cheng Z. (2020). Cross-sectional studies: strengths, weaknesses, and recommendations. Chest.

[bib0044] Winning L., Lundy F.T., Blackwood B., McAuley D.F., El Karim I. (2021). Oral health care for the critically ill: a narrative review. Crit. Care.

[bib0045] Zaitoun R.A., Said N.B., de Tantillo L. (2023). Clinical nurse competence and its effect on patient safety culture: a systematic review. BMC. Nurs..

